# Antioxidant activity, total polyphenol, anthocyanin and benzyl-glucosinolate contents in different phenotypes and portion of Japanese Maca (*Lepidiummeyenii*)

**DOI:** 10.1016/j.heliyon.2024.e32778

**Published:** 2024-06-10

**Authors:** Harumi Uto-Kondo, Yuna Naito, Masaya Ichikawa, Rio Nakata, Akifumi Hagiwara, Koji Kotani

**Affiliations:** a Department of Bioscience, Nihon University, 1866 Kameino, Fujisawa-shi, Kanagawa, 252-0880, Japan; b Department of Bioscience in Daily Life, Nihon University, 1866 Kameino, Fujisawa-shi, Kanagawa, 252-0880, Japan; c Japan VegeMaca Association., 6-15-10-304 Tsukiji, Chuo-ku, Tokyo, 104-0045, Japan; d Department of Global Coexistence Studies, Nihon University, 1866 Kameino, Fujisawa-shi, Kanagawa, 252-0880, Japan

**Keywords:** Maca (*Lepidium meyenii*), Phenotype, Antioxidant, Anthocyanin, Benzyl-glucosinolate

## Abstract

Maca (*Lepidium meyenii*), mainly grown in Peru, is a traditional herbal medicine that is mostly used to improve sperm motility and serum hormone levels. Maca phenotypes are represented by purple, black, yellow, white, and mixed colors. Recently, a method for Maca cultivation has been established in Japan. Therefore, we determined the effects of different phenotypes and portions on the antioxidant activities and total polyphenols, anthocyanins, and benzyl-glucosinolate contents in Japanese Maca. Purple Maca skin possessed the highest contents of both total polyphenols, antioxidant activity and anthocyanin content in all Macas. Regarding the benzyl-glucosinolate content, white maca had the highest content and was not correlated with antioxidant activity. In the present study, we revealed that purple Maca skin is recommended for high polyphenol content, antioxidant activity, and anthocyanin content. The results of this study will be useful for selecting phenotypes for the improvement of antioxidant activity or hormone balance.

## Highlights

• We first time determined the effects of different phenotypes and three types of portions of Japanese Maca on the antioxidant activities and total polyphenol, anthocyanin, and benzyl-glucosinolate content.
• Purple Macas possessed high contents of both total polyphenols and antioxidant activities due to anthocyanin.
• In particular, the skin of deep purple Maca had the highest in Macas.
• The antioxidant activity was not correlated with benzyl-glucosinolate content.

## 1 Introduction

Maca (*Lepidium meyenii*), mainly grown in Peru, is a traditional herbal medicine used to improve physical endurance in male impotence and female hormonal imbalances [1,2]. The major consumer countries for Maca-based products include the USA, Canada, the UK, Germany, China, Japan, and the Netherlands [3]. Maca grows at altitudes between 2800 and 5000 m above sea level. Plants adapt well to extremely harsh high altitude conditions [4]. Recently, a method of Maca cultivation has been established in Fukushima in Japan for over 30 years. Maca phenotypes are represented by purple, black, yellow, white, and mixed colors [5,6]. In adult male rats, yellow Maca and black Maca improved epididymal sperm count, and the three phenotypes of Maca increased the sperm count in the vas deferens, indicating that Maca acts as a modulator of sperm count at the reproductive tract level [7]. In ovariectomized female mice, black Maca appears to have a more beneficial effect on latent learning than yellow Maca and red Maca [8]. Benzyl-glucosinolate, a maca-specific component, contributes to these effects and has attracted attention.

The nutritional value of maca only partially lies in its major dietary constituents, which include starch, dietary fiber, and protein. A variety of compounds with pharmacological and nutritional significance in maca include non-starch polysaccharides, polyphenols, malamedas, macaenes, macamides, glucosinolates, and macahydantoins [9,10]. Among the antioxidants that are beneficial to health, polyphenols are one of the most important because of their antioxidant and anti-inflammatory actions, and because they directly and indirectly present antimicrobial, antiallergic, and anti-atherogenic properties [11,12]. In addition, many studies have shown that polyphenols counteract cellular oxidative stress by modulating physiological enzymes and receptor activities, thus preventing oxidative stress-related diseases [13].

Sun Y et al. reported that essential oil, lipid and polysaccharide in black Maca showed higher effect than yellow and red phenotype [14]. Although it has been reported that black Maca powder has the antioxidant capacity to scavenge free radicals and protect cells against oxidative stress [15], it remains unclear whether the Maca phenotype and portion affect antioxidant activity. In addition to varietal and color differences, the total polyphenol content of agricultural products is affected by the plant parts. In the present study, Japanese Maca with different phenotypes were divided more precisely into seven colors (purple, black, yellow, white, and their mixture), although most of the experiments that have been performed to classify maca by color have been based on the three colors of red, white, and black. On the other hand, the skin of various plants has a color and high total polyphenol content [16]. Anthocyanin is a type of polyphenol that has purple color. Purple-colored skin contain more anthocyanins than other polyphenols [20]. However, there are no reports that have measured the antioxidant capacity of polyphenols in different phenotypes and portions (whole, skin, and pulp) in Maca. Furthermore, there is no information on the anthocyanin fraction found in maca. We therefore investigated to understand the portion in antioxidant activity, total polyphenol, anthocyanin. Furthermore, we studied the benzyl-glucosinolate contents and its correlation with the antioxidant activity.

## 2 Materials and methods

### 2.1 Maca samples

**Fig. 1:**
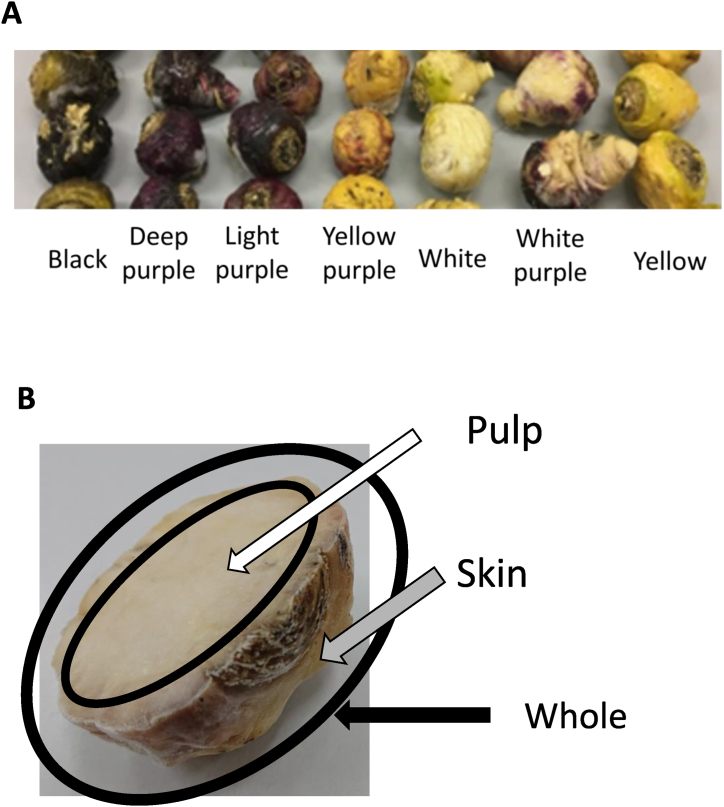
Seven Maca of different phenotypes and portions (A) phenotypes and (B) portions.

Maca roots ware used as Maca. The seven colors of Maca are represented by colors labelled purple, black, yellow, white, and their mixture. All the Macas grown in Fukushima in Japan were purchased from the Japan Vege Maca Association (Tokyo, Japan). All Maca after cutting off the leaves with similar sizes and shapes (63.6 ± 15.8 g weight, 5.4 ± 0.4 cm length, 5.1 ± 0.7 cm diameter) were selected and cutted the Macas radially from the center so that they were even. Each Maca was separated into whole, skin, and pulp (whole without skin) (Fig. 1).

### 2.2 Chemicals and reagents

1,1-diphenyl-2-picryl-hydrazyl (DPPH), ascorbic acid (ASA), catechin, ethanol, and cyanidin-3-glucoside were purchased from Wako Pure Chemical Industries (Osaka, Japan). Phenol reagent was purchased from Nacalai Tesque Inc. (Kyoto, Japan).

### 2.3 Preparation of sample solution for antioxidant activity, total polyphenol and anthocyanin content

To determine the antioxidant activity and total polyphenol content, all Macas after cutting radially was chopped and mixed with ethanol (four times the weight of Maca) for 2 min in a centrifuge tube using a food mixer. The sample was then incubated at room temperature for 15 min and centrifuged (3000 rpm, 5 min, 4 °C). The sample solutions were stored at −80 °C until analysis.

### 2.4 Determination of antioxidant activity

The antioxidant activity was measured using the DPPH radical-scavenging activity method. DPPH traps the hydrogen of a phenol compound, causing the DPPH radical to disappear [17]. The radical scavenging activities of the seven Maca colors were spectrophotometrically measured using the DPPH radical. Extraction of Maca of different colors was mixed with 2 mL of ethanol containing DPPH (0.1 mmol/L). The solution was then shaken and incubated at 37 °C for 20 min in the dark. The decrease in the absorbance of DPPH was measured at 516 nm using a UV-1280 UV-VIS spectrophotometer (Shimadzu Corporation, Kyoto, Japan). The total polyphenol content was calculated using a calibration curve prepared using ASA.

### 2.5 Total polyphenol content

The total polyphenol content was determined using the Folin-Ciocalteu colorimetric method [18]. The phenol reagent (0.1 mL) was added to the sample solution (0.1 mL). After the solution was allowed to stand for 60 min at room temperature, the absorbance at 760 nm was measured using a UV-1280 UV-VIS spectrophotometer. The total polyphenol content was calculated using a calibration curve prepared using catechin.

### 2.6 Anthocyanin concentration

Singh et al. reported that the molar absorbance of anthocyanin-3-glucosides calibration curves for the absorbance read at 520 nm for the absorbance read at each compound maximum absorbance (*λ*_max_) showed good linearity with a coefficient of correlation of 0.999 or higher [19]. Therefore, we determined the concentrations of anthocyanin in Macas by total light absorption at 520 nm using a UV-1280 UV-VIS spectrophotometer. Anthocyanin concentration was calculated using cyanidin-3-glucoside.

### 2.7 Benzyl-glucosinolate quantification

Benzyl-glucosinolate was analyzed using high-performance liquid chromatography (HPLC) with a photodiode array [20] at the laboratory of the Biodynamic Plant Institute Co. Ltd. *(Hokkaido, Japan).* Each sample was single analyzed using pooled Macas which had the same weight cut radially from the center (n = 6). Intact glucosinolates were extracted from using a methanol-water mixture at high temperatures to disable myrosinase activity. After sulfatase treatment, the desulfoglucosinolates were eluted with water, and the eluate was freeze-dried. The residue was collected using an exact volume of water. Detection and quantification were achieved by comparing the retention times and UV spectra of the commercial reference standards.

### 2.8 Statistical analyses

Data represent the mean values ± SD for weight, length, diameter, polyphenol content, antioxidant activity and anthocyanin content. All statistical analyses were performed using Excel for Microsoft 365 MSO, Version 2405 (Microsoft Corp., Redmond, WA, USA). Statistical significance was evaluated by two-way analysis of variance (ANOVA) followed by Tukey's test. Different letters in the same row indicate significant differences (P < 0.05).

## 3 Results and discussion

**Fig. 2:**
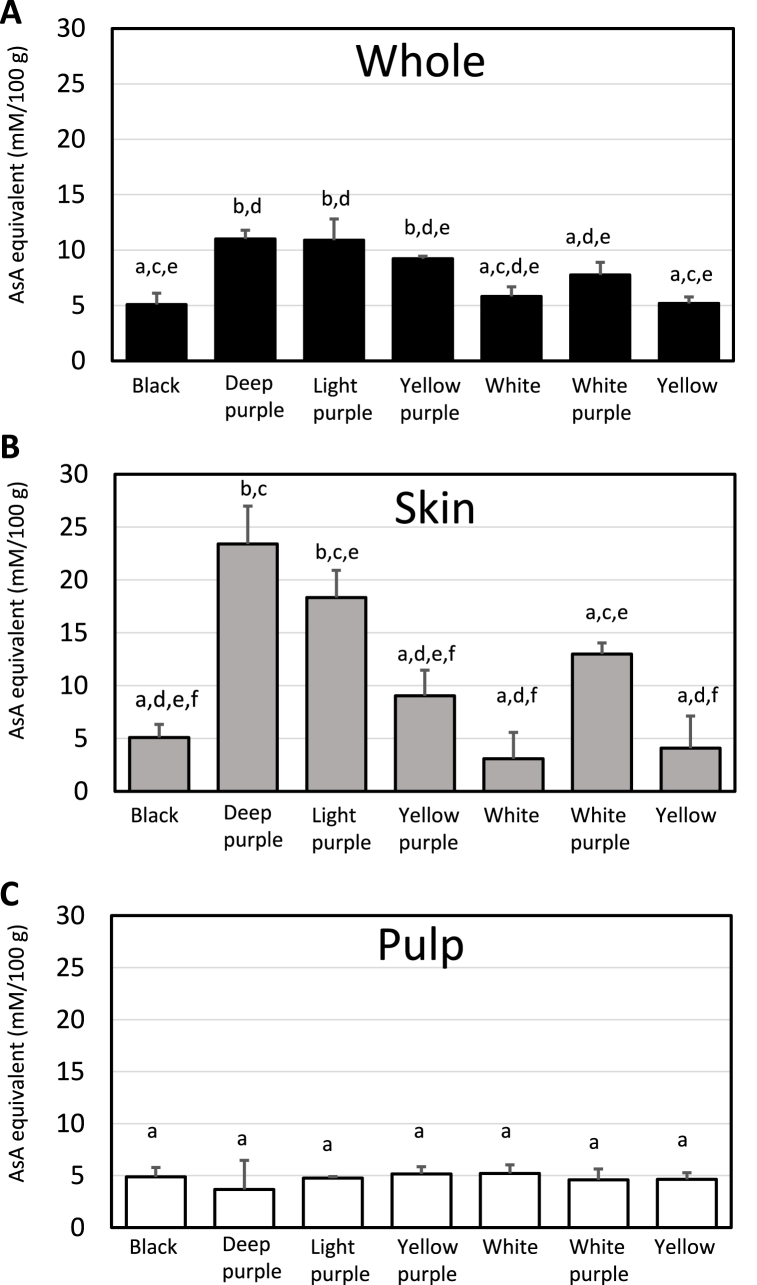
DPPH radical scavenging activity of different phenotypes and portions of Maca (A) whole, (B) skin and (C) pulp. Data represent mean the values ± SD; n = 3. The significance of the differences was calculated using Tukey's test. Different letters in the same row indicate significant differences (P < 0.05).

**Fig. 3:**
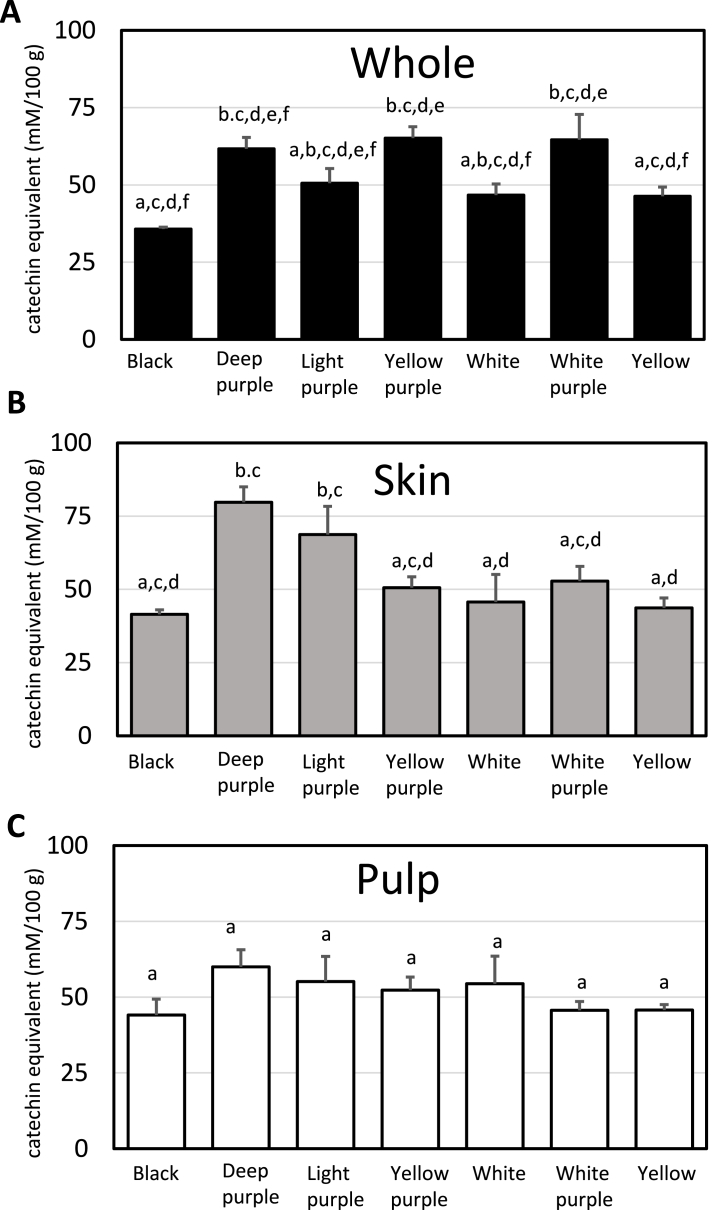
Total polyphenol content of different phenotypes and portions of Maca (A) whole, (B) skin and (C) pulp of seven phenotypes of Maca. Data represent mean the values ± SD; n = 3. The significance of the differences was calculated using Tukey's test. Different letters in the same row indicate significant differences (P < 0.05).

The antioxidant activity and total polyphenol content of Japanese Maca according to the seven different colors and three different parts are shown in Fig. 2, Fig. 3.

As shown in Fig. 2A, the antioxidant activity and total polyphenol content of whole Maca were higher in deep purple (11.0 mmol/L), light purple (10.9 mmol/L), yellow purple (9.2 mmol/L), and white purple (7.8 mmol/L) than in Black, White and Yellow. Likewise, the antioxidant activity of Maca skin was higher in Deep purple (23.4 mmol/L), Light purple (18.3 mmol/L) Yellow purple (13.0 mmol/L), and White purple (9.0 mmol/L) compared to other three color samples (Fig. 2B). From the results shown in Fig. 2A and B, it is thought that Maca, which is purple in color, contains a relatively large amount of total polyphenols. Indeed, the amount of polyphenol of whole Maca was higher in purple Macas; yellow purple (65.1 mmol/L), white purple (64.6 mmol/L), deep purple (61.7 mmol/L), light purple (50.6 mmol/L) compared to white (46.7 mmol/L), yellow (46.4 mmol/L), and black (35.8 mmol/L) (Fig. 3A). Consistent with the antioxidant activity of Maca skin in Fig. 2B, the amount of total polyphenol of Maca skin is the highest in deep purple (79.7 mmol/L), followed by light purple (68.7 mmol/L), and followed by white purple (52.8 mmol/L), yellow purple (50.5 mmol/L), white (45.7 mmol/L), yellow (43.6 mmol/L), and black (41.5 mmol/L). Purple Maca skin contains a large amount of polyphenols and is thought to have antioxidant activity. Neither black nor yellow nor white Maca significantly contributed to the total polyphenol content. In contrast, there was no significant difference in the antioxidant activity and total polyphenol content between Maca pulps (Fig. 2, Fig. 3C). From the above results, it was concluded that purple is the major bioactive substance affecting antioxidant activity and total polyphenol content, and the deeper color of purple and its skin contains more total polyphenol. Anthocyanins are purple and are one of the most abundant groups of naturally occurring polyphenols present in many flowers, fruits, vegetables, and grains [21]. Anthocyanins have antioxidant [22,23] and anti-inflammatory properties [24].

The skin of various fruits and vegetables has a deep color and a high total polyphenol content [16]. In particular, purple colored skin may contain more anthocyanins than other polyphenols [25]. The Zutphen Elderly Study demonstrated that a flavonoid (another phenolic acid derivatives)-rich diet reduced the rate of atherosclerosis *in vivo* [26,27]. This led to the hypothesis that coffee exerts its anti-atherosclerotic effects via anti-oxidative and/or anti-inflammatory functions [[28], [29], [30]].

**Fig. 4:**
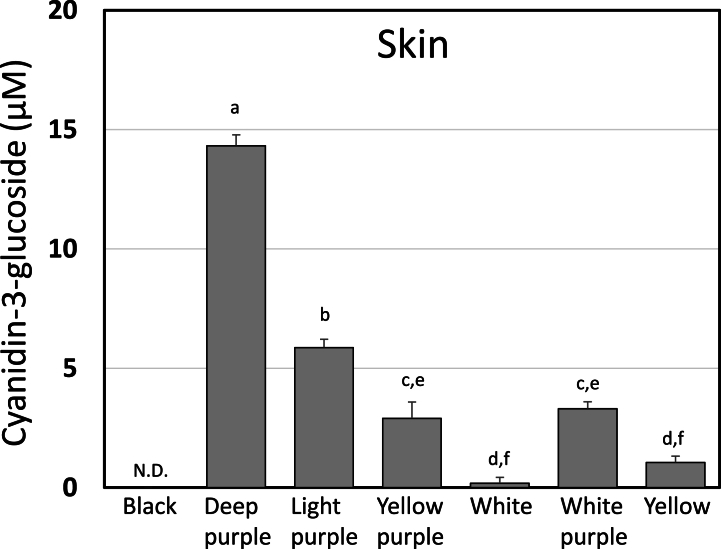
Anthocyanin content of different phenotypes of Maca skin Data represent mean the values ± SD; n = 3. The significance of the differences was calculated using Tukey's test. Different letters in the same row indicate significant differences (P < 0.05).

Therefore, we measured the anthocyanin content in the Maca skin (Fig. 4). Deep purple Maca (14.3 μmol/L) contributed the highest amount of the total polyphenol and anthocyanin, followed by light purple (5.9 μmol/L), white purple (3.3 μmol/L), yellow purple (2.9 μmol/L), yellow (1.1 μmol/L), white (0.2 μmol/L) or black (N.D.). Neither black nor yellow nor white Maca contributed significantly to the total polyphenol content, owing to their anthocyanin content (Fig. 4).

**Fig. 5:**
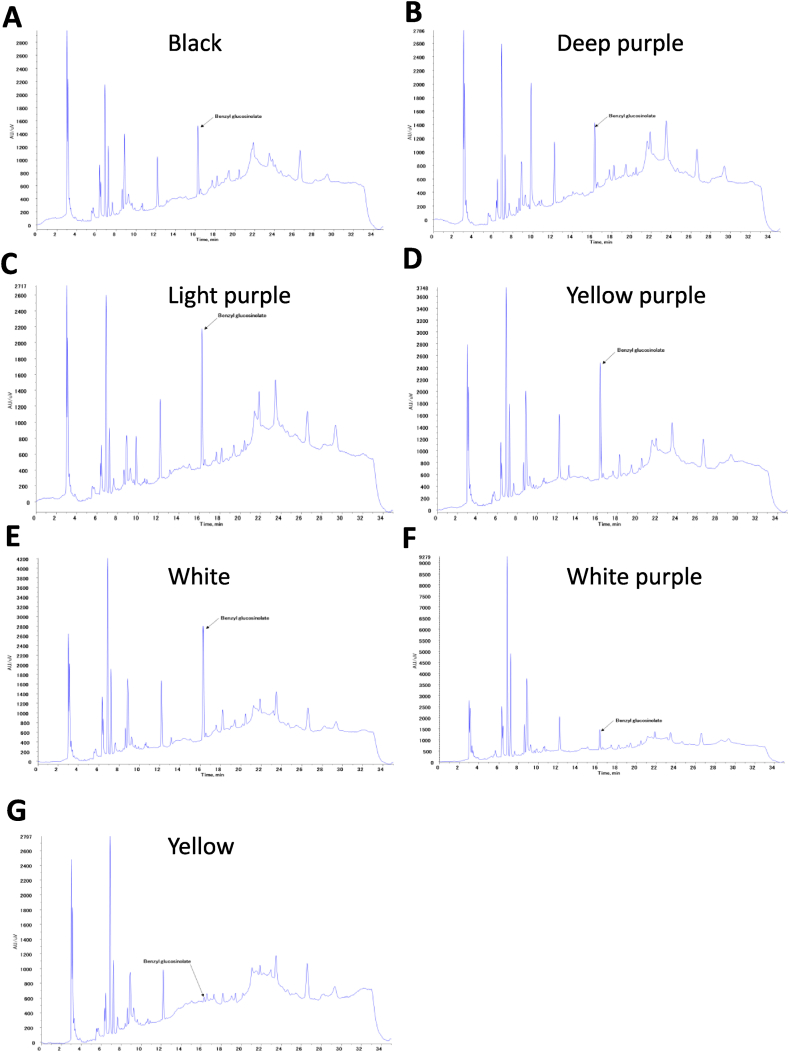
**HPLC chromatograms of benzyl-glucosinolate of different phenotypes of whole Mac**a (A) black, (B) deep purple, (C)light purple, (D)yellow purple, (E)white, (F)white purple and (G)yellow.

In contrast, Maca is rich in benzyl glucocinolates, which improve physical endurance of male impotence and female hormonal imbalances. Finally, we determined the benzyl-glucosinolate content by HPLC analysis of the whole different phenotype Maca (Fig. 4A–G and Tagle.1). Benzyl-glucosinolate content (100 g) was highest in white (8.86 mg), followed by yellow-purple (7.54 mg), light-purple (7.25 mg), black (4.37 mg), deep-purple (4.00 mg), white-purple (2.97 mg), and yellow (0.19 mg). The average number of Macas was 4.99 mg. Yan et al. reported that total glucosinolates showed DPPH radical scavenging capacity at concentrations ranging from 1 to 4 mg/mL, and BGC showed little antioxidant activity [31]. All Maca pulps had no color and no significant differences in antioxidant activity or total polyphenol content (Fig. 2, Fig. 3C). Based on these results, we concluded that there was no correlation between color, antioxidant activity, and amount of benzyl-glucosinolate (Fig. 5).

## 4 Conclusions

Here, we showed, for the first time, that different phenotypes and portions of Japanese Maca, purple Maca has polyphenol content and antioxidant activity due to anthocyanin. The present study therefore sheds new light on the potential anti-atherogenic properties of Maca, in addition to improving physical endurance, for male impotence and female hormonal imbalances.

## Data availability

The authors confirm that the data supporting the findings of this study are available within the article and its supplementary materials.

## CRediT authorship contribution statement

**Harumi Uto-Kondo:** Writing – review & editing, Writing – original draft, Visualization, Validation, Supervision, Software, Resources, Project administration, Methodology, Investigation, Formal analysis, Data curation, Conceptualization. **Yuuna Naito:** Writing – original draft, Methodology, Investigation, Formal analysis, Data curation. **Masaya Ichikawa:** Writing – original draft, Methodology, Investigation, Formal analysis, Data curation. **Rio Nakata:** Writing – original draft, Methodology, Investigation, Formal analysis, Data curation. **Akifumi Hagiwara:** Supervision, Resources. **Koji Kotani:** Supervision, Resources.

## Declaration of competing interest

The authors declare that they have no known competing financial interests or personal relationships that could have appeared to influence the work reported in this paper.
